# Editorial Notes

**Published:** 1894-02

**Authors:** 


### Editorial Notes.

Dr. W. H. Inghram and Miss Loyala McAfee, both of this city, were married on February 5th.

Dr. Thomas D. McKown, of Chicamauga, Ga., spent several days in the city last week, and favored us with a pleasant call.

The Congress of the American Physicians and Surgeons will be held in Washington, D. C., on May 29th, 31st and June 1st, 1894.

Dr. J. B. Pendergrass, of Jefferson, Ga., and Miss Watie Heath were united in marriage January 31st. THE RECORD extends heartiest congratulations to the happy couple.

We regret to learn of the death of Dr. C. J. Carter, of Lake Park, Ga. Dr. Carter was a progressive citizen and stood high in the profession as a man of much ability, and his high character and genialities made him many warm friends.

SIXTH ANNUAL MEETING.—The sixth annual meeting of the Tri-State Medical Society of Alabama, Georgia and Tennessee will be held in Atlanta, Ga., Tuesday, Wednesday and Thursday, October 9th, 10th and 11th, 1894. The proposition to change the name to the "Southeastern Medical Society," will be considered. This will embrace the territory east of the Mississippi and south of the Ohio.

MRS. H. V. M. MILLER, wife of Dr. Miller, died January 8th. Following are resolutions passed by students of Atlanta Medical College:

ATLANTA, GA., January 9th, 1894.

WHEREAS, In the exercise of his loving Providence, the Omniscient God has seen fit to take to himself the estimable wife of

Dr. H. V. M. Miller, thus severing that sacred bond of companionship enjoyed by them during so many years of happiness and great usefulness; therefore be it

*Resolved* 1. That we, the students of the Atlanta Medical College, extend hereby our condolence to our beloved dean and professor in this hour of greatest bereavement.

2. That we wish for him solace to his sorrowing heart and strength to his feeble frame in order that he may yet spend years of usefulness in the pursuit of that noble calling which he has so nobly filled.

3. That a copy of these resolutions be sent to Dr. Miller, published in the daily papers of the city, in the *Atlanta Medical and Surgical Journal,* and in THE SOUTHERN MEDICAL RECORD.

M. G. CAMPBELL, H. M. SMITH,

T. C. JEFFORDS, W. J. SHAW,

J. L. SANBORN, Committee.

---

### THE ELEVENTH INTERNATIONAL MEDICAL CONGRESS.

### IMPORTANT NOTICE.

Gentlemen who comtemplate attending the Eleventh International Medical Congress which meets at Rome, March 29th to April 5th, will not be able to secure the reduced rates offered by steamship lines and European railroads unless with the following documents:

1st. General certificate securing the reduction over the North German Lloyd steamship line from New York and return over the railroads of Italy, Russia, France, Spain, Portugal, Belgium and England, good for two months (March 1st to April 30th) in Europe.

2d. A *Lettre d' Invitation* required only to secure reductions on French railways.

3d. A railway pass (*Carta di riconoscinento*) consisting of three coupons required for the Italian railways.

These documents, with full instructions relative to steamship and railroad rates itinerary of various journeys in Europe, tariff regulations and concessions, etc., will be furnished on application to

CHARLES A. L. REED, M. D.,

Member of the American Committee of the Eleventh International Medical Congress, 487 West Sixth street, Cincinnati, O.

